# Assessment and Comparison of the Efficacy of Methotrexate, Prednisolone, Adalimumab, and Tocilizumab on Multipotency of Mesenchymal Stem Cells

**DOI:** 10.3389/fphar.2020.01004

**Published:** 2020-07-03

**Authors:** Shuang Liu, Takeshi Kiyoi, Marina Ishida, Masaki Mogi

**Affiliations:** ^1^ Department of Pharmacology, Ehime University Graduate School of Medicine, Toon, Japan; ^2^ Department of Advanced Research Support Center, Ehime University, Toon, Japan

**Keywords:** mesenchymal stem cells, antirheumatic drugs, chondrogenesis, cartilage regeneration, xenografted model

## Abstract

Mesenchymal stem cell (MSC)–based articular regeneration might be beneficial for both protecting and rebuilding cartilaginous tissues in the management of rheumatoid arthritis. However, it is unclear how current immunosuppressive strategies influence the multipotency of MSCs. The present study was undertaken to profile the direct effectiveness of major antirheumatic drugs including methotrexate, prednisolone, adalimumab, and tocilizumab on the multipotency of MSCs, with a special focus on chondrogenesis. The inhibitory effects of methotrexate on adipogenesis, osteogenesis, and chondrogenesis were observed to occur in a dose-dependent manner in an *in vitro* differentiation system. Prednisolone enhanced adipogenesis, but reduced alkaline phosphatase activity in osteoprogenitors and suppressed the formation of chondrospheroids. Adalimumab suppressed alkaline phosphatase activity, while tocilizumab diminished osteogenesis and chondrogenesis of MSCs *in vitro*. Chondrogenesis of antirheumatic drug-treated MSCs was also evaluated *in viv*o using a scaffolded spheroid-engrafted murine model. The biologics examined appeared to be relatively safe for cartilaginous formation, but methotrexate and prednisolone exhibited opposing influences on chondrogenesis. Taken together, these results reveal the direct efficacy of major antirheumatic agents on the multipotency of MSCs. Therefore, our findings suggest that optimization of medication protocols is further required for therapeutic approaches involving cartilaginous tissue engineering.

## Introduction

Rheumatoid arthritis (RA) is characterized by persistent and chronic synovitis in the joint, followed by spontaneous progressive joint destruction, which is evident on X-ray as erosion and progressive narrowing of joint spaces (Sharp et al., 1985). Because joint destruction directly causes joint pain and functional disability, the basic strategy of RA management is altering the systemic immune status to prevent or delay joint destruction. However, after a prolonged period of time, the damage is almost irreversible and very limited options remain other than trying to prevent further destruction of the joint and considering prosthetic replacement arthroplasty. Recently, treatment with disease-modifying antirheumatic drugs (DMARDs) has achieved outstanding results for systemic inflammatory management. However, even when clinical remission is achieved, cartilage damage may continue to progress. With this in mind, approaches to protect against structural damage and repair existing damage have emerged for joint regeneration.

Several therapeutic strategies have been applied for regeneration of joint destruction, such as oral or intravenous medications, treatment of a particular joint with intra-articular injection, and surgical intervention. However, the high proliferative capacity and osteogenic/chondrogenic capabilities of mesenchymal stem cells (MSCs) have catapulted them to the forefront of cell-based therapy for joint-destructive diseases (El-Jawhari et al., 2014; Vizoso et al., 2019). Clinical induction of self-repair capacity *via* transplantation of multipotent MSCs under strong inhibition of synovitis has been attempted in several previous studies (Ito et al., 2018). In an effort to improve the clinical efficacy of these therapies, techniques for MSC-based tissue engineering have also been developed for the establishment of *in vitro*–formed spheroids (Zubillaga et al., 2020).

Although the concept of MSC-based joint regeneration has demonstrated promising results and opens new therapeutic possibilities for RA treatment, obstacles for its use are not trivial. For example, the proliferative and chondrogenic potential of RA patient-derived MSCs is reduced compared with normal MSCs. Moreover, RA patient-derived MSCs exhibit decreased cellular telomere length, which may be associated with the patient’s age, background, DMARDs treatment, and altered expression of genes implicated in local adhesion and cell cycle pathways (Kastrinaki et al., 2008). The microenvironment and its interaction with the delivered cell population seem to be crucial for maximizing the therapeutic potential of MSCs. Most patients with RA have been treated and are undergoing continuously treatment with methotrexate, corticosteroids, and/or biologics. However, whether these immunosuppressors can affect the multipotency of transplanted MSCs or their capacity to repair cartilage or bone damage is unknown.

Understanding how individual antirheumatic drugs influence MSCs is necessary for future joint regeneration approaches. The present study was undertaken to screen for direct effects of individual antirheumatic agents on the multipotency of human bone marrow-derived MSCs, using an *in vitro* differentiation system. With a special focus on chondrogenic differentiative capacity, we also evaluated the *in vivo* effects of these agents in a murine MSC chondrospheroid-engrafted model.

## Materials and Methods

### Cell Culture and Multipotency Characterization of MSCs

Human cartilage–derived primary MSCs were purchased from the Japanese Collection of Research Bioresources Cell Bank (JCRB, Osaka, Japan). These cells exhibit an expanded lifespan by carrying *bmi-1*, *E6*, *E7*, and *hTERT* genes, as previously described (Mori et al., 2005). According to instructions from JCRB, MSCs were cultured in preconditioned Poweredby10 Medium (GlycoTechnica, Yokohama, Japan). All experiments were performed with passage 8–13 MSCs.

For observation of MSC multipotency, a Human Mesenchymal Stem Cell Functional Identification Kit (R&D Systems, Minneapolis, MN) was used according to manufacturer’s instructions (Liu et al., 2019). MSCs were differentiated in adipogenic, osteogenic, or chondrogenic conditioned medium, with or without application of the antirheumatic agents methotrexate, prednisolone, adalimumab, and tocilizumab at the indicated concentrations. To evaluate adalimumab, 1 ng/ml (58.82 nM) recombinant human tumor necrosis factor α (TNF-α; PeproTech, Rocky Hill, NJ) was added to each kind of differentiation medium, to establish an inflammatory microenvironment following an established protocol (Žigon-Branc et al., 2018).

Briefly, for adipogenic differentiation, MSCs were cultured in adipogenic differentiation medium containing adipogenic supplements hydrocortisone (0.5 μM), isobutylmethylxanthine, and indomethacin. Seven days later, MSCs were stained with an anti-mouse fatty acid binding protein (mFABP) antibody and counterstained with Hoechst^®^ 33342 (0.5 μg/ml). For fluorescence quantification, image acquirement was performed in an imaging chamber using MetaXpress software (Molecular Devices, Tokyo, Japan; (Kiyoi et al., 2018). Thirty-two fields were captured in each well with 100–400 ms exposure times at a magnification of ×200. The integrated intensity of each image was determined under the background-subtracted condition. For functional analysis, lipid droplets that had formed during adipogenic differentiation were stained in fixed cells using an Oil Red O stain kit (Abcam, Cambridge, UK) according to the manufacturer’s instructions.

For osteogenic induction, MSCs were cultured in osteogenic differentiation medium containing dexamethasone (10 nM), ascorbate-phosphate, and β-glycerophosphate for 21 days. Subsequently, surface and functional markers of induced osteocytes were observed. For detection of osteocalcin, cells were labeled using a primary anti-human osteocalcin antibody (R&D Systems, #967801). Image-capturing methods were similar to those described above for adipogenic detection. Functional activity of alkaline phosphatase (ALP) was detected on day 14 in MSCs undergoing osteogenic differentiation using an ALP staining kit (Wako, Tokyo, Japan) according to the manufacturer’s protocol.

For experiments using MSCs spheroids without any scaffold, 2.5 × 10^5^ MSCs were pelleted in chondrogenic differentiation medium supplemented with dexamethasone (100 nM), ascorbate-phosphate, proline, pyruvate, recombinant tumor growth factor-β3, insulin, transferrin, selenious acid, bovine serum albumin, and linoleic acid. After 21 days of induction, formed spheroids were sectioned on a cryotome and subjected to aggrecan and CD44 detection. Images were captured using a fluorescence microscope (BZ-9000; Keyence, Osaka, Japan). The volume of formed macromass was quantified by magnetic resonance imaging (MRI) acquired under a three-dimensional (3D) T2-weighted flash sequence protocol using an MRmini SA110 scanner (DS Pharma Biomedical, Osaka, Japan). Coronal and sagittal images were collected and reconstructed to obtain the volume of chondrospheroids.

### Preparation of Chondrospheroids in a 3D Collagen Matrix

To establish an *in vivo* chondrogenic drug-screening system, MSCs were differentiated into chondrospheroids on a 3D collagen matrix, which was prepared by immersing a round shape Honeycomb sponge (Koken, Tokyo, Japan) in 50 μl of 2% Atelocollagen Implant (Koken) in a round-bottom 96-well culture plate. The matrix was incubated at 37°C for 1 h, after which 200 μl of prewarmed Poweredby10 Medium was added to the well. The formed matrix was calibrated at 37°C in a humidified atmosphere with 5% CO_2_ overnight. MSCs (2.5 × 10^5^) were seeded on plates in chondrogenic differentiation medium and pelleted for chondrogenic differentiation. After 21 days of induction, the formed chondrospheroids were harvested and ready for animal transplantation. To detect chondrogenic makers, formed spheroids were sectioned on a cryotome and subjected to aggrecan and CD44 detection. Images were captured using a fluorescence microscope.

### Establishment of a Xenograft Human RA Model

Peripheral blood, synovium, articular cartilage, and bone explants were obtained from RA patients who had undergone prosthetic replacement arthroplasty for therapeutic purposes. A murine xenograft RA model was established as previously described (Liu et al., 2017). Briefly, explants of synovium, articular cartilage, and bone obtained from RA patients were grafted onto the muscle at the level of the fourth to sixth lumbar vertebra of male NOD/ShiJic-scid (NOD/SCID) mice aged 6–10 weeks (CLEA Japan, Tokyo, Japan). The same patient-derived peripheral blood monocytes (5 × 10^6^) were resuspended in 100 μl serum and intraperitoneally implanted into mice immediately after the grafting surgical procedure. For MSC-derived chondrospheroid transplantation, predifferentiated spheroids with or without scaffold were implanted on the surface of damaged RA patient-derived cartilage.

Eight weeks after transplantation, mice were sacrificed by cervical dislocation. Implanted tissues were removed from xenograft mice and subjected to fixation, decalcification, and paraffin embedding. The invasion of synovium into cartilage was observed on hematoxylin-stained sections by light microscopy.

### *In Vivo* Chondrogenic Drug-Screening Model

Predifferentiated chondrospheroids in a 3D collagen matrix were implanted into NOD/SCID mice. After the subcutaneous tissue was exposed under anesthetic conditions, the oblique external abdominal muscle was scraped with a scalpel until it bled. The chondrospheroids were then implanted. Healthy donor-derived peripheral blood monocytes (5 × 10^6^) were intraperitoneally implanted into mice.

One week after transplantation, antirheumatic regents were continuously subcutaneously infused into mice using implanted osmotic Alzet pumps (DURECT, Cupertino, CA).

For clinical RA treatment, after three oral weekly doses of 2 mg methotrexate, a steady-state plasma concentration of methotrexate (approximately 39.9 ng/ml) is achieved (Inoue and Yuasa, 2014). A steady-state concentration of 3 μg/ml adalimumab is achieved by subcutaneously injection of 40 mg adalimumab every 2 weeks (Ternant et al., 2015). For tocilizumab, the steady-state plasma concentration is 12.3 μg/ml after 8 mg/kg intravenous injection every 2 weeks. Maintenance therapy with a relatively low dose (≤10 mg/day) of prednisolone is used for RA treatment. A dose-calibrated C_max_ of 20.7±6.5 μg/L/1 mg for oral administration and 88.3±24 μg/L/1 mg for intravenous injection have been reported (Czock et al., 2005). By referring to the results of these pharmacokinetic studies and previous *in vivo* studies using murine arthritis models, an equivalent dose for mouse administration based on body surface area was calculated, and an effective dosage range to suppress the systemic inflammatory status with minimum side effects was chosen for each antirheumatic regent (Nair and Jacob, 2016). The doses were 0.25 or 0.5 mg/kg for methotrexate (Singh et al., 2019), 0.1 or 0.2 mg/kg for prednisolone (Hofkens et al., 2011), 0.75 or 1.5 mg/kg for adalimumab (Zafir-Lavie et al., 2017), and 4 or 8 mg/kg for tocilizumab (Thiolat et al., 2014). Mice implanted with saline-filled pumps were used as controls.

Four weeks later, chondrospheroids were explanted from cervically dislocated mice. The volume of formed macromass was quantified by MRI as described above for *in vitro* studies. Explants were then fixed using 4% paraformaldehyde and subjected to histological analysis. Expression of aggrecan and CD44 in spheroids was detected using an immunofluorescence staining technique.

### Statistical Analysis

All experiments were designed in a completely randomized multifactorial format. Sample distributions were analyzed using the Kolmogorov-Smirnov test. Dose-response titrations were fitted by nonlinear regression. Results are expressed as the mean ± standard error of the mean (SEM). The Mann-Whitney *U*-test was used for nonparametric interclass comparisons and the results are described by the median and interquartile range (difference between 25^th^ and 75^th^ percentiles). The Wilcoxon signed-rank test was used for paired comparisons and the results are expressed as the mean ± standard deviation (SD). P < 0.05 was considered significant. All data were analyzed using Prism 8 (GraphPad Software, La Jolla, CA).

## Results

### Effects of Antirheumatic Agents on Adipogenic Differentiation Potential of MSCs

Multipotency was evaluated in antirheumatic agent-treated MSCs. Adipocyte protein 2, also called FABP4, was used as a surface marker for differentiated adipocytes. In addition, the formation of lipid droplets, which were stained by Oil Red O, was also observed as a functional marker of differentiated adipocytes.

**Figure 1:**
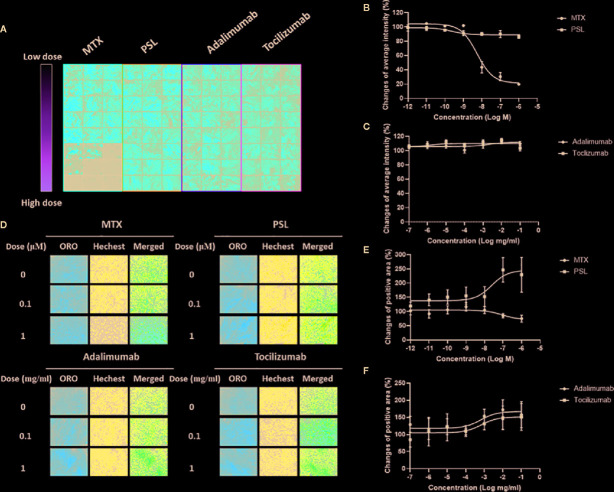
Effect of MTX, PSL, adalimumab, and tocilizumab on MSC differentiation into adipocytes. **(A)** Typical imaging screening panel for quantification of mFABP4 expression. MSCs were seeded on 96-well plates, and 32 fields were captured in each well using a high-throughput image quantitation system. One of 32 fields is shown. **(B,** **C)** The titration curve of mFABP4 expression in MSCs treated with **(B)** (MTX or PSL, and **(C)** adalimumab or tocilizumab. The average change of fluorescent intensity was obtained from 96 images for each concentration. Results are presented as mean ± SEM. **(D)** Typical images of lipid droplets analysis in antirheumatic drug-treated MSCs. Following Oil Red O staining, lipid droplets present as red-stained areas. **(E, F)** Changes in positive area of lipid droplet in MSCs treated with **(E)** MTX or PSL, and **(F)** adalimumab or tocilizumab. The red-stained area was calibrated by the number of nuclei. Results are presented as mean ± SEM. mFABP4, mouse fatty acid binding protein; MSC, mesenchymal stem cell; MTX, methotrexate; PSL, prednisolone; SEM, standard error of the mean.

A typical screening panel for quantification of FABP4 expression in antirheumatic agent-treated MSCs is shown in **Figure 1A**. A total of 32 panels were obtained, and changes in the average fluorescence intensity of each sample were evaluated. According to the titration curves, FABP4 expression in methotrexate-treated MSCs decreased in a dose-dependent manner with an *EC_50_* of 54.53 nM, whereas prednisolone exhibited a slightly inhibitory effect on FABP4 induction (**Figure 1B**). Direct application of adalimumab or tocilizumab did not elicit any effect on FABP4 expression in MSCs (**Figure 1C**). Fluorescence imaging of lipid droplets revealed that prednisolone treatment increased lipid droplet formation (**Figures 1D–F**). Treatment with >100 nM methotrexate suppressed droplet formation. Both adalimumab and tocilizumab elevated lipid droplet induction at concentrations between 100 ng/ml and 0.1 mg/ml. Taken together, treatment with methotrexate suppressed the adipogenic differentiation potential of MSCs, while prednisolone, adalimumab, and tocilizumab increased lipid droplet induction of MSCs.

### Methotrexate, Adalimumab, and Tocilizumab Downregulated Osteogenic Differentiation

**Figure 2:**
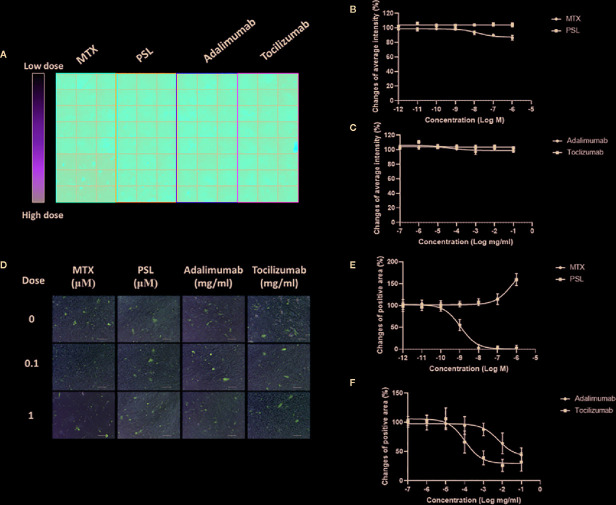
Osteogenic differentiation potencies of antirheumatic drug-treated MSCs. **(A)** Typical imaging screening panel for quantification of osteocalcin expression. One of 32 screening panels is shown. **(B**, **C)** The titration curve of osteocalcin expression in MSCs treated with **(B)** MTX or PSL, and **(C)** adalimumab or tocilizumab. Average change of fluorescent intensity was obtained from 96 images for each concentration. Results are presented as mean ± SEM. **(D)** Typical images of ALP-activity analysis in antirheumatic drug-treated MSCs. Positive areas present as purple-stained areas (200×; scale bar: 40 μm). **(E**, **F)** Changes of relative positive area of ALP-activity assay in MSCs treated with **(E)** MTX or PSL, and **(F)** adalimumab or tocilizumab. The purple-stained area was segmented from the background and the change of relative area was quantified. More than four fields per section and an average of five sections for each concentration were used for semiquantitative analysis. Results are presented as mean ± SEM. ALP, alkaline phosphatase; MSC, mesenchymal stem cell; MTX, methotrexate; PSL, prednisolone; SEM, standard error of the mean.

Populations of osteogenic cells positive for osteocalcin, bone-specific ALP, or both have been identified (Long et al., 1995). Treatment of MSCs with prednisolone, adalimumab, or tocilizumab did not affect the expression of osteocalcin, a noncollagenous protein of the bone matrix specifically synthesized by osteoblasts that is often used as a marker for the bone formation process (Dirckx et al., 2019) (**Figures 2A–C**). Treatment with high concentrations of methotrexate (more than 100 nM) slightly downregulated osteocalcin expression in MSCs. Activity of osteoprogenitor-derived ALP, which sensitively indicates new bone formation and osteoblast activity (Abe et al., 2019), exhibited different induction patterns compared with osteocalcin (**Figures 2D–F**). Methotrexate, adalimumab, and tocilizumab inhibited ALP activity in osteoprogenitors in a dose-dependent manner. *EC_50_* values were 11.03 nM for methotrexate, 607 ng/ml for adalimumab, and 10.64 ng/ml for tocilizumab. In contrast, prednisolone elevated ALP activity at concentrations higher than 1 μM.

### Methotrexate, Prednisolone, and Tocilizumab Inhibit Chondrospheroid Formation *In Vitro*

**Figure 3:**
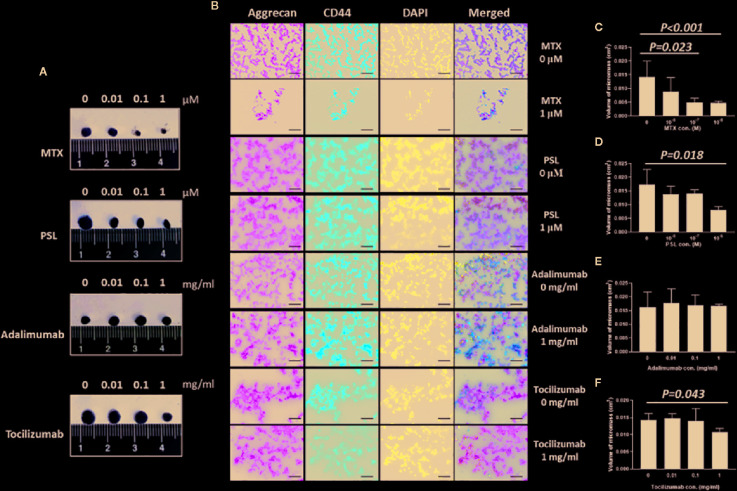
*In vitro* chondrogenic assessment of antirheumatic agent-treated MSC-spheroids. **(A)** Micromass formation by antirheumatic agent-treated spheroids after 28 days of induction culture. **(B)** Typical images of aggrecan and CD44 expression in MSC spheroids. Fixed micromasses were embedded and sectioned on a cryotome. Expression of aggrecan (green) and CD44 (red) were observed using immunohistochemical staining (200×; scale bar: 40 μm). **(C–F)** Quantification of volume of micromass formed by chondrospheroids treated with **(C)** MTX, **(D)** PSL, **(E)** adalimumab, and **(F)** tocilizumab. MRI imaging of micromass was acquired under a three-dimensional T2-weighted flash sequence protocol; coronal and sagittal images were collected and reconstructed to obtain the volume of chondrospheroids. Results are expressed as the mean ± standard deviation (SD) (n=5). MSC, mesenchymal stem cell; MRI, magnetic resonance imaging; MTX, methotrexate; PSL, prednisolone.

For chondrospheroid generation, MSC spheroids were cultured *in vitro* using a high cell-density pellet culture system, which mimics the cellular condensation requirement for embryonic mesenchymal chondrogenesis and provides the physical and biochemical environmental factors conductive to cartilage formation. The spheroids exhibited as compact rounded cell aggregates that maintained their physical structure for the duration of induction and, finally, formed detectable micromasses (**Figure 3A**). Differentiation levels of the chondrospheroids were assessed by histological staining using the chondrogenic surface marker CD44 and aggrecan, also known as cartilage-specific proteoglycan core protein, as a functional marker of chondrogenesis. Chondrospheroids developed a multilayered matrix-rich morphology (**Figure 1B**). CD44-positive cellular aggregates and an aggrecan-rich extracellular matrix were observed, regardless of whether antirheumatic agents had been applied.

As an indicator of cell proliferation, matrix deposition, and intercellular space volume, the volume of chondrospheroids was quantified (Martinez et al., 2008) (**Figures 3C–F**). Compared with nontreated chondrospheroid, volumes were decreased to 34.84% in 100 nM methotrexate-treated chondrospheroids (*P* = 0.023) and 33.63% in 1 μM methotrexate-treated chonderspheroids (*P* < 0.001). Significantly decreased volumes were also observed in prednisolone-treated (1 μM) and tocilizumab-treated (1 mg/ml) chondrospheroids. Taken together, methotrexate, prednisolone, tocilizumab exhibited greater or lesser inhibitory effects on *in vitro* chondrospheroid formation.

### Profiling the Effects of Antirheumatic Agents on Chondrogenesis of MSCs *In Vivo*

**Figure 4:**
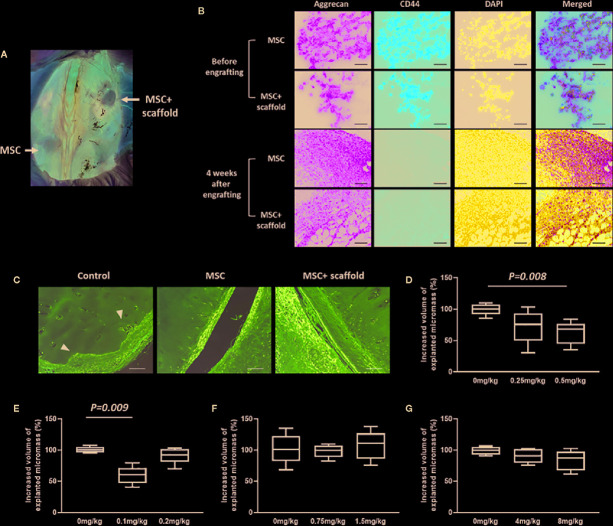
Establishment an *in vivo* chondrogenic drug-screening system. **(A)** Cartilaginous mass formation in chondrospheroid-engrafted mice. Chondrospheroids with and without scaffold were implanted to NOD/SCID mice preengrafted with healthy-donor derived PBMCs. Four weeks later, cartilaginous particles had formed. **(B)** Typical images of aggrecan and CD44 expression in MSC spheroids before engrafting and 4 weeks after chondrospheroid transplantation. Expression of aggrecan (green) and CD44 (red) were observed using immunohistochemical staining (200×; scale bar: 40 μm). **(C)** Cartilage regenerative capability of chondrospheroids. Predifferentiated chondrospheroids were transplanted into a human xenografted RA model. Implanted RA-patient derived synovium, cartilage, and bone were explanted 8 weeks after chondrospheroid transplantation. Hematoxylin and eosin staining was performed on sections; typical images are shown (200×; scale bar: 20 μm; arrow: synovial invasion to cartilage). **(D–G)** Quantification of volume of cartilaginous particles formed in mice treated with **(D)** MTX, **(E)** PSL, **(F)** adalimumab, or **(G)** tocilizumab. Antirheumatic agents were continuously subcutaneously infused for 30 days in chondrospheroid-engrafted mice. MRI imaging of explanted micromass was acquired to evaluate the volume of chondrospheroids. Results are described as the median and interquartile range (difference between 25^th^ and 75^th^ percentiles) (n=5). MSC, mesenchymal stem cell; MTX, methotrexate; PBMCs, peripheral blood mononuclear cells; PSL, prednisolone.

With the aim of evaluating the effects of antirheumatic agents on MSC-based joint regeneration, a chimeric human-mouse model was established for *in vivo* drug screening. To increase the viability and adhesion of chondrospheroids prepared for *in vivo* transplantation, a porous 3D collagen matrix was used as a scaffold. Chondrospheroids with and without scaffold were implanted into NOD/SCID mice preengrafted with healthy donor-derived PBMCs. Four weeks later, a firm cartilaginous mass was formed by chondrospheroids with scaffolds, while a softer and more unstable cartilaginous mass was formed by chondrospheroids without scaffolds (**Figure 4A**). Cartilaginous particles were then explanted and histologically characterized (**Figure 4B**). Cartilaginous particles with and without scaffolds formed a densely layered structure with aggrecan-rich extracellular matrix. Compared with chondrospheroids before engrafting, CD44 expression, which was confirmed on cell surfaces in chondrospheroids before engrafting, became undetectable.

The capability of MSCs-derived spheroids for cartilage repair was examined in a xenografted RA model. In this model, RA patient-derived synovial tissue, bone, and articular cartilage were xenografted into NOD/SCID mice. According to the results of histological evaluation, with an inflammatory microenvironment supported by autologous PBMC engraftment, the patient-derived synovium invaded into implanted cartilage and even bone tissue, whereas synovial invasion was markedly inhibited by chondrospheroid transplantation (**Figure 4C**). Especially in chondropheroids of scaffold-transplanted mice, the regeneration of cartilage tissue and repair of damaged cartilage surface were observed. These results indicated that MSC-derived chondrospheroids can survive in a xenografted murine model and are capable of repairing articular cartilage defects. Thus, chondrospheroids of scaffold-implanted mice are useful for further drug efficacy assessments.

After subcutaneous delivery of antirheumatic agents, the volumes of explanted cartilaginous mass were quantified (**Figures 4D–G**). Compared with untreated mice, methotrexate (10 mg/kg) significantly decreased cartilaginous mass formation (*P* = 0.041). Interestingly, the inhibitory effect of prednisolone was only observed in the 5 mg/kg prednisolone-treated mice (*P* = 0.009), but not 10 mg/kg prednisolone-treated mice. The biologics did not show any influence on cartilaginous mass formation.

## Discussion

The management of rheumatology faces an enormous challenge: the largely irreversible nature and joint damage and erosion. Current immunosuppressive agents achieve neither restoration of joint architecture nor reversal of damage (Kerrigan and McInnes, 2020). Although MSCs might be beneficial for both protecting and rebuilding cartilaginous tissues, how current immunosuppressive strategies influence the multipotency of MSCs is largely unknown. The present study was undertaken to profile the direct effectiveness of major antirheumatic drugs, including glucocorticoids, conventional synthetic DMARDs, and biologics, on the multipotency of MSCs. By acknowledging pharmacological, metabolic, and cellular mechanisms, we attempt to provide insights into the contribution of these drugs with regard to healing capacity and MSC-based tissue engineering.

As a first-line drug for the treatment of RA, nearly all patients with inflammatory arthritis are currently given methotrexate in the absence of contraindications (Cronstein and Aune, 2020). If patients do not respond, they are prescribed other immunosuppressive therapies, such glucocorticoids or biologics, usually in combination with methotrexate. Methotrexate functionally inhibits the *de novo* synthesis of purines and pyrimidines that is required for DNA and RNA synthesis, as well as cell proliferation. In our study, *in vitro* treatment with methotrexate significantly suppressed the multilineage (adipogenic, osteogenic, and chondrogenic) potencies of MSCs, consistent with the results of a previous study (Beane et al., 2016). Undifferentiated stem cells exhibited resistance to methotrexate; even concentrations of methotrexate as high as 50 μM did not affect cell proliferation. However, once stem cells were placed in a differentiation-inducing environment, their tolerance level was significantly decreased, as a 5- to 10-fold higher susceptibility was observed in nonstem status cells. These changes might result from a dihydrofolate reductase-mediated mechanism.

In addition to direct application *in vitro*, continuous subcutaneous infusion of methotrexate also markedly inhibited the formation of cartilaginous masses in MSC spheroid-grafted mice. Compared with oral administration, enhanced bioavailability can be achieved by constant long-term subcutaneous delivery, which can prolong the potency of polyglutamated methotrexate (the active form of methotrexate) in tissues (Kremer et al., 1986; Herman et al., 1989). Although better responses to methotrexate or other antirheumatic drugs can be achieved by parenteral administration, this route imparts a higher risk of effects on the multipotency of MSCs, which should be considered when protecting and rebuilding cartilaginous tissues. In addition, whether the chondrogenic potency of MSCs can be restored by concomitant administration of folic acid or folinic acid should be examined to optimize cartilage tissue engineering for RA treatment.

In individuals with RA, oral glucocorticoid usage continues to be widespread, although it is potentially declining (Hardy et al., 2020). As glucocorticoids are required for both adipogenesis and osteoblastogenesis, their influence on the multipotency of MSCs should be evaluated. In *in vitro* culture systems, dexamethasone is important for the initiation of adipogenic differentiation by activating C/EBPδ expression through binding to intracellular glucocorticoid receptor (Chen et al., 2016). For osteoblastogenic differentiation, dexamethasone promotes cell proliferation, resulting in the induction of ALP activity. These hypotheses were confirmed by the results of our *in vitro* assessment, including the enhancement of lipid drop formation and elevation of ALP activity in prednisolone-treated MSCs. However, exposure to supraphysiological levels of glucocorticoids could be associated with reduced bone mineral density, impaired bone quality, and increased risk of fracture. Shifting the balance from osteoblastogenesis to adipogenesis has been widely accepted as one of the mechanisms by which glucocorticoids diminish osteoblast differentiation (Hartmann et al., 2016).

Interestingly, during chondrogenic differentiation, detrimental effects were observed in 1 μM prednisolone-treated MSCs and 0.1 mg/kg prednisolone-treated mice. However, this inhibitory effect disappeared in 0.2 mg/kg prednisolone-treated mice. The impact of glucocorticoid exposure on chondrogenesis of MSCs seems ambiguous with the combination of this apparently contradictory evidence. First, dexamethasone is used in conjunction with transforming growth factor beta-1 (TGF-β1) in our chondrogenic differentiation system for the induction of differentiation of cartilage derived-MSCs. A previous study reported that the influence of steroids is dependent on the source of cells (tissue of origin and its microenvironment) (Shintani and Hunziker, 2011). In this study, dexamethasone enhanced TGF-β1-induced chondrogenesis in bone marrow-derived MSCs, but significantly inhibited TGF-β1-induced synovium-derived aggregates. The results of our *in vitro* assessment revealed that exposure to prednisolone suppressed the chondrogenic capacity of cartilage-derived MSCs. Second, at present, we can offer no plausible explanation for the variable effects of prednisolone on *in vivo* cartilage-like tissue formation of MSCs. Corticoids can reportedly cause cartilage hypertrophy during MSCs differentiation (Kovermann et al., 2019). By detecting a genetic hypertrophic marker, the ratio of *COL2A1/COL10A1*, researchers found that exposure to dexamethasone led to hypertrophic chondrogenesis, which is associated with a temporary cartilage template that will remodel into bone. Based on these results, they suggested that to obtain a more stable chondrogenic induction, the availability of glucocorticoids should be reappraised. We evaluated the chondrogenic capability of chondrospheroids by assessing the volume of *in vivo* formed micromass and quality of cartilaginous mass, as evaluated by aggrecan expression. However, no hypertrophic marker was included in the *in vivo* screening system, thus we do not yet know whether cartilage-like tissues in 0.2 mg/ml prednisolone-treated mice formed stable cartilage-like tissues or hypertrophic phenotypes. For further usage aimed at quality control of cartilaginous masses in the field of regenerative medicine, optimization of our i*n vivo* drug evaluation system is probably required, such as the addition of a panel to monitor the expression levels of cartilaginous genes. Taken together, as a result of extreme high bioavailability, the influence of glucocorticoids on articular self-repair and regeneration are unignorable in some RA patients with long-term maintenance therapy, even at low dosages.

Compared with the universal effects of small molecular antirheumatic drugs, biologics are target-specific immunosuppressors. With regard to regenerative capability, the TNF inhibitor adalimumab had little impact on chondrogenesis of MSCs *in vitro* or *in vivo*, but inhibited ALP activity in a dose-dependent manner without disrupting osteocalcin expression in osteogenesis. ALP is expressed during early bone development and in calcifying cartilage, and is often observed on the cell surface and in matrix vesicles (Golub et al., 1992). Later during differentiation, ALP activity is reduced while osteocalcin expression is upregulated. The observed decrease of ALP activity may indicate that osteoprogenitors at an ‘early differentiation stage’ are diminished by adalimumab.

Interleukin-6 (IL-6) is a pleiotropic cytokine that mainly activates the STAT3 signaling pathway to promote downstream gene transcription. It was previously reported that autocrine/paracrine IL-6 signaling of MSCs contributes to chondrogenic differentiation (Kondo et al., 2015), as well as osteogenic differentiation (Xie et al., 2018). In the present study, inhibition of IL-6-mediated signaling by tocilizumab *in vitro* markedly reduced ALP activity during osteogenesis and the formation of macromass during chondrogenesis of MSCs. However, within the therapeutic dosage range, tocilizumab did not present obvious opposing effects on cartilaginous mass formation *in vivo*. Both adalimumab and tocilizumab appeared to be relatively safe for approaches involving MSC-based cartilage regeneration.

In addition to the influences of antirheumatic drugs on dominant potential factors for articular regenerative, the effects of these drugs on adipogenic induction is of interest. Although it has been hypothesized that synovial adipocytes may contribute to maintenance of articular energy metabolism and the stem cell niche, the precise role of adipocytes in the regulation of cartilage homeostasis remains poorly understood (Labusca and Zugun-Eloae, 2018). Direct application of methotrexate downregulated the expression of FABP4 in a dose-dependent manner, whereas prednisolone only slightly inhibited FABP4 induction. This supports the results of a computational analytical approach using publicly available synovial tissue transcriptomic datasets (Wang et al., 2019). A significantly decreased fraction of adipocytes in synovium of RA patients, who had received a long-term methotrexate or prednisolone treatments, was found compared with the healthy controls. However, the inhibitory effects of methotrexate and prednisolone on lipid droplet formation were different. Prednisolone even enhanced the capability of adipogenic cells to form lipid droplets for storage. Dramatic functional compensation of epidermal FABP (FABP5), which is also expressed in adipocytes, may provide an explanation for the disconnect between the FABP4 differentiation readout and lipid droplet formation. Because FABP5 compensates for the function of FABP4 deficiency in overall metabolic homeostasis, the antirheumatic agent-induced inhibitory effects may have been compensated by FABP5 (Furuhashi and Hotamisligil, 2008). A study to dissect the expression balance and address the potential adipogenic effects of these FABP isoforms may provide further understanding of the role of MSC adipogenic potency in articular homeostasis maintenance.

In summary, we profiled the influence of major antirheumatic drugs on the multipotency of human cartilage–derived MSCs that are an important cell source for tissue renewal and regeneration. While biologics exhibited relatively little effects, small molecular compounds, such as methotrexate and prednisolone, somewhat affected the osteogenesis and chondrogenesis of MSCs. In clinical management of RA, glucocorticoids and methotrexate are generally considered to be a part of the initial treatment strategy, and therapy using these drugs is often started as soon as the diagnosis of RA is made. Additionally, in patients responding insufficiently to synthetic chemical compounds, biological agents are always commenced with methotrexate. While expecting remarkable immunomodulatory efficacy, the opposite effects on self-repair should always be considered by rheumatologists, especially in the case of using a high-dose of methotrexate or a combination of glucocorticoids and methotrexate for long-term treatment. Thus, optimization of medication protocols is required for treatment approaches aimed at protecting and rebuilding cartilaginous tissues. Notably, the most common treatment strategies rely on a combination of conventional synthetic DMARDs, targeted synthetic DMARDs, and biological DMRDS instead of single agent administration. The present profile is not sufficient to justify cross-strategy management of medications and tissue engineering for RA therapy and additional investigations are required to provide a complete perspective of antirheumatic drugs in the regenerative field.

## Conclusion

According to the results of the present study, biologics appear to be relatively safe for cartilaginous formation, although methotrexate and prednisolone exhibited opposing influences. Therefore, our findings suggest that optimizing medication protocols is required for therapeutic approaches involving cartilaginous tissue engineering.

## Data Availability Statement

The raw data supporting the conclusions of this article will be made available by the authors, without undue reservation, to any qualified researcher.

## Ethics Statement

The studies involving human participants were reviewed and approved by Ethics Committee of Ehime University School of Medicine. The patients/participants provided their written informed consent to participate in this study. The animal study was reviewed and approved by University Committee for Animal Research, Ehime University.

## Author Contributions 

SL: conceptualization, methodology, formal analysis, writing—original draft, funding acquisition. TK: methodology, investigation, writing, review and editing, validation. MI: investigation. MM: supervision, project administration.

## Funding

SL was supported by the Japan Society for the Promotion of Science (JSPS) and Grants-in-Aid for Scientific Research (KAKENHI) grant number 18K08389.

## Conflict of Interest

The authors declare that the research was conducted in the absence of any commercial or financial relationships that could be construed as a potential conflict of interest.
